# Learning aptitude, spatial orientation and cognitive flexibility tested in a virtual labyrinth after virtual stress induction

**DOI:** 10.1186/s40359-015-0080-5

**Published:** 2015-07-05

**Authors:** Marcel Delahaye, Patrick Lemoine, Shanique Cartwright, Gunnar Deuring, Johannes Beck, Marlon Pflueger, Marc Graf, Henning Hachtel

**Affiliations:** Universitaere Psychiatrische Kliniken (UPK) Basel, Wilhelm Klein Str. 27, 4012, Basel, Switzerland

**Keywords:** Stress, TSST, Virtual Reality, Learning aptitude, Spatial orientation, Cognitive flexibility

## Abstract

**Background:**

Under stressful conditions such as in an emergency situation, efficient information processing is essential for reasonable responses.

Purpose of the Study: Virtual Reality (VR) technology is used to induce stress and to test three main cognitive functions for decision making in stressful situations.

**Methods:**

A VR task was developed to induce stress following the Trier Social Stress Test (TSST) protocol and two VR cognitive performance tests to measure learning aptitude, spatial orientation and cognitive flexibility. Participants (N = 31) gave a public speech in front of a virtual audience (TSST) and later had to find their way out of different VR labyrinths. The first exercise tested spatial orientation and learning aptitude where participants had to learn aspects of the ground layout and geometric icons had to be identified as correct in order to be able to exit. The second labyrinth tested cognitive flexibility on the background of the Wisconsin Card Sorting Test.

Statistical tests: Correlations were analyzed using Kendall Tau Correlation (One-tailed tests with p set to 0.05 for all analyses). Heart rate (HR) was calculated from the RR time values and averaged across the TSST- speech and the post-stress period. Autonomic nervous system reactivity was defined as the deviation of HR during TSST- speech condition from post-stress baseline measurement. A repeated-measures *t*-test was used to analyze differences.

**Results:**

The newly developed virtual stress test was successfully adapted from the original TSST. Participants perceived the task as stressful and scored an average of 5.7 points on a 1–8 Likert Scale. As a physiological stress parameter, increased heart rates of the participants showed that they were more stressed during the TSST procedure compared to the post-stress period. Also, the subjective stress perception, has a strong correlation with the results of the cognitive tasks performed after the stress induction.

**Conclusions:**

The more a participant experienced the TSST as stressful, the lower their learning aptitude and spatial orientation were found to be at the end of the study. On the other hand, if someone perceived the virtual TSST as “unexpected”, as an indicator for a mild stress response, their cognitive flexibility was improved.

Potential Implications: The findings indicate that both, the VR stress induction scenario using TSST, as well as the VR cognitive tests, are a first successful step towards a better ecological validity in neuropsychological testing.

## Background

Literature analysis has shown (Chaytor and Schmitter-Edgecombe 2003) that conventional neuropsychological tests have a limitation in predicting everyday cognitive skills and performance. Beside developing Virtual Reality (VR) programs to test cognitive functions of learning aptitude, spatial orientation and cognitive flexibility in an innovative way using complimentary 3D-Powerwall software, one major goal of this paper is to demonstrate changes in thinking or cognitive functions and rational decision making under acute stress. The background of the study was the 7th Framework EU Project SAVE ME (Grant agreement no.: 234027). In that project, documented human behavioral patterns in stressful situations had to be identified to develop appropriate guidelines for evacuation planning. A new approach in our study (which was part of the EU project) is that both stress induction as well as cognitive performance tests are given in a VR setting to investigate how stress affects behavior, but using an artificial environment that can still achieve high ecological validity.

Acute stress arises if a subject perceives a self-discrepancy between demands and pressures of the recent past and anticipated demands and pressures of the near future. The focus of our research is on acute rather than chronic stress. In reviewing literature on how acute stress affects cognition and performance, it soon becomes clear that the constellation of factors triggering a stress response differs situationally and individually (Dickerson and Kemeny 2004; Kaluza 2004). Mainly these influential factors include intrinsic versus extrinsic motivation, free will versus being forced to act under stress and the physiological state of the participant. The quality of the task itself - is it life-threatening or just a daily hassle, as well as the level of expertise of the participant, the locus of control, or perception of predictability, the participant’s coping style and stress vulnerability all affect stress responses. Other considerations include the participant's vulnerability to stress, the task duration and stressor type. For example, is the stressor noise, heat, time constraints or, psychosocial? A clear deficit in all preceding studies aiming to get a better understanding of how stress influences cognition is the variation of how researchers induced stress. Therefore one of the aims of this study is to create a standardized way of inducing stress. A review of current literature findings are summarized below concerning learning aptitude, spatial orientation, cognitive flexibility and decision making with the common parameter of them all taking place under stress.

### Learning aptitude in an acutely stressful situation

Working memory plays an important role in learning aptitude (Chan et al. 2011) and seems to be less available for retrieving information during periods of stress (Wolf 2006). Also memory retrieval (Kuhlmann et al. 2005), verbal episodic memory (Newhouse et al. 2010), working memory as well as accuracy in an “n-back paradigm” (Schoofs et al. 2008) are significantly impaired after the Trier Social Stress Test (TSST). Hancock and Warm 1989 induced cognitive stress by increasing room temperature, and describes the effect of this as a form of decreased attention, which has a negative impact on learning aptitude.

In contrast other studies concluded that stress can also facilitate learning and memory processes. Joëls et al. 2006 found that acute stress, induced with different stressors such as the Water-Maze labyrinth, examination stress or other physical stressors, improved memory when the memory acquisition phase and stressor shared the same spatiotemporal context. For example, if there was context-congruency where learning material is associated in time and space with the stressor. Smeets et al. 2007 found that psychosocial stress induced using the TSST, may strengthen the consolidation of memory material when the stressor matches the ‘to be learned’ material in place and time.

### Spatial orientation in an acute stressful situation

Spatial orientation is another aspect that is affected during a stressful situation. Duncko et al. 2007 showed that Cold Pressor Test (CPT) stressed participants performed better in localizing a hidden platform in a virtual navigation Morris water task compared to their non-stressed counterparts. Currently there is a clear lack of knowledge about the role of stress on spatial orientation in humans, as most studies have only been undertaken in animals (Duncko et al. 2007 Yang et al. 2003 found that in rats, the effect of stress on spatial memory can be switched from impairment to enhancement depending on both the stress experience as well as the age of the subject. de Quervain et al. 1998 showed that stress decreases spatial memory tasks ability during a water-maze in rats when the stressor, electrical foot-shock, was administered 30 min before retention testing but had no influence when the electrical foot-shock was administered 2 min or 4 h before testing. Other results on rats showed a throughout decline in spatial orientation after acute cold stress (Stillman et al. 1998).

### Cognitive flexibility after stress

Cognitive flexibility, which is elementary for problem solving (Beversdorf et al. 1999), is significantly impaired after exposure to psychosocial stress induced by the TSST (Alexander et al. 2007). Another finding also suggests that auditory stress of 90 dB of white noise impairs cognitive flexibility (Hillier et al. 2006). Snyder 2013 found that strategy set-shifting performance, which is described as a special form of cognitive flexibility, is reduced in adult rats which were socially and chronically stressed during their adolescence. During that study, stress on the animal was induced with the “resident-intruder paradigm” during which the animal is positioned in the cage of another animal or group of animals of the same species, in a way that permits a non-lethal conflict.

### Role of stress on decision-making

According to Baddeley (2012) decision-making can be regarded as a higher level cognitive function, which is composed of the interaction of different basic cognitive components such as visuo-spatial tracking and cognitive flexibility. One general and well documented finding of the interaction between stress and decision-making states that strong emotional stress induces a ‘fight or flight’ condition, in which perception, learning aptitude, cognitive flexibility and reasoning are affected in a way that enables the individual to react quickly (Cannon 1915; Hilton 1982). During the stressful situation, the individual shifts to a simpler mode of information processing and decision making that may help to focus on the threat (Kowalski-Trakofler et al. 2003). A similar phenomenon is called perceptual tunneling which describes that the peripheral field of attention is decreased and the focus remains primarily on the stressor (Ritter et al. 2007).

Decision-making appears to be affected by acute stress, in that stressed participants tend to consider more high risk decisions (Pabst et al. 2013). Entin and Serfaty 1990 tested decision-making skills of military personnel under time constraints in a combat simulation study. Untrained military commanders exhibited difficulty in maintaining an accurate image of tactical situations, and made less effective decisions under conditions of high stress and uncertainty. Also Wickens et al. 1989 found that stress that was induced by administering an additional cognitive task during flight simulation for pilots, negatively affected their decision making capacity. Heilman et al. 2003 quotes Martindale and Greenough 1973 that white-noise induced stress lowers the capacity of innovative problem solvingSchwabe and Wolf 2009 showed that when a subject is stressed, they revert to previous ineffective solutions to the same problem at the expense of choosing an unknown but better option available to them. For example, stressed subjects evaluated via the Socially Evaluated Cold-Pressor Test: SECPT remained significantly longer with a poor choice compared to controls. Farhadbeigia et al. 2012 stated that it is not yet understood as to the degree of which different levels of stress impairs decisions.

Collectively, the findings on the effects of acute stress on cognitive functions and decision-making leave it open as to what degree stress enhances or decreases cognitive functions in an emergency situation.

Another problem with the current findings is the low external and ecological validity. As stressors can vary from noise or cold temperatures to psychosocial stress, it remains difficult to generalize conclusions from each individual study. A second point of criticism with respect to ecological validity is that the design in conventional neuropsychological tests is very academic and abstract. For example, subjects may have to learn word lists. Chaytor and Schmitter-Edgecombe 2003 stated that many neuropsychological tests have only a moderate level of ecological validity when predicting everyday cognitive functioning. Chaytor and Schmitter-Edgecombe 2003 main argument is that there is only little empirical evidence that the same neuropsychological tests that were developed for clinical diagnosis can be used to evaluate real-world functioning. Chaytor and Schmitter-Edgecombe 2003 introduced the concepts of verisimilitude and veridicality. Verisimilitude is the degree to which the cognitive demands of a neuropsychological test are similar to the cognitive demands in the everyday environment. Veridicality indicates the degree to which an existing test is empirically related to measures of everyday functioning. As both aspects are typically low in existing tests, the conditions for transferring findings to predict cognitive performance in a real stressful emergency situation are suboptimal. With the VR technique, the conditions of a real emergency scenario are able to be artificially simulated but with a feeling of authenticity (immersion). The participants should experience a situation similar to their being in a real emergency.

The current study has two main goals, one of which is first to test the impact of stress on cognitive functions in a VR setting that feels realistic and authentic on one hand but is standardized and therefore equal for every subject on the other hand. This could be an innovative approach for future cognitive testing and a cost-effective way for stress induction. And the second goal is to acquire a better understanding of the influence of stress on the individual components of decision making including learning aptitude, spatial orientation and cognitive flexibility.

## Methods

### Subjects

31 healthy volunteers (Caucasians; 12 female, 19 male, age 25.1 ± 6.6), all of whom were unrelated individuals of Swiss-German descent, with no clinical psychiatric diagnosis were recruited via flyer for ten weeks at the University of Basel. The text was: “Ever wondered if Virtual Reality can be stressful? Contact us!”.

### Procedure

#### **•**Stress induction using Virtual TSST

Psychosocial stress is used for stress induction. Psychosocial stress, although a specific form of stress, still falls under the general definition of stress from Salas et al. 1996 It states”stress is a process by which certain work demands evoke an appraisal process in which perceived demands exceed resources and result in undesirable physiological, emotional, cognitive and social changes.”

The Trier Social Stress Test (TSST) from Kirschbaum et al. 1993 is a well-known psychological procedure that allows experimenters to induce stress under laboratory conditions. In the Trier Social Stress Test, participants have to deliver a public speech in front of an audience. Originally, actors were used to form this audience and induce social stress. It has been proven that the TSST is an effective tool to induce stress and to stimulate the hypothalamic-pituitary-adrenal (HPA) axis (Dickerson and Kemeny 2004).

**Fig. 1:**
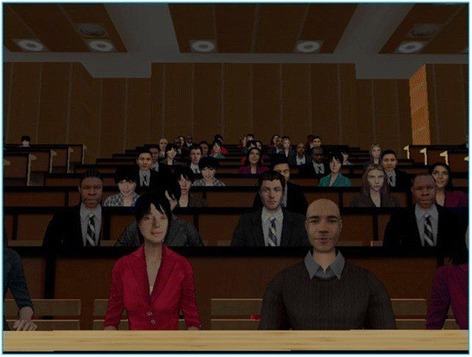
Screenshoot of the Virtual Lecture Hall

In the present study, a VR adaptation of the TSST was developed. After a two minute preparation, the participants introduce themselves for a 5 min period, as though they are in a job interview for a position as a ‘Professor for Ethics’ or as a ‘referee’. Their introduction is based on their educational background. This takes place in a virtual lecture hall in front of a virtual audience. The main focus of the public speech had to be on personal, and not academic strengths. Different studies have already demonstrated that the TSST can also be induced by Virtual Avatars (Fich et al. 2014 Jönsson et al. 2010 Kelly et al. 2007). In these studies three avatars (Fich et al. 2014 Jönsson et al. 2010), and later, five avatars (Kelly et al. 2007) were used. In the present study, the virtual audience consisted of 80 avatars. The authors chose this high number of avatars to create more stress due to the increased number of people, and to reduce the likelihood that the participants could focus only on one avatar, which they might find non-realistic or even distracting. The avatars in the test were programmed with a 3D-animation technique and their individual level of noise and movement vary following a fixed sequence. To imitate a typical public audience and create a highly immersive experience, expressive faces and natural, precisely chosen gestures were selected for the human avatars. In the beginning, the audience has very little movement and the noise level is low. Towards the end of the experiment, the noise level rises and the avatars start to move more frequently to increase the stress level. The virtual audience is mixed regarding gender and ethnic groups but for the most part are Caucasian. The test is given in a Virtual Environment (3 sided power wall; size: 3 m × 1.5 m each). Fig. 1

#### **•**Spatial orientation and learning aptitude test in Virtual Reality (VR)

Spatial orientation is the basic process for navigation. It consists of extracting information, forming mental representations, and using that representation for route planning (Darken et al. 1998). Ruddle et al. 2000 state that particularly for mental representations of space, “cognitive maps” (Howard and Kerst 1981 can be regarded as essential for navigating a virtual environment. In the current study, two sets of VR cognitive performances tests or labyrinths were developed. In the first cognitive VR test, spatial orientation and learning aptitude were tested. This is referred to as the Memory Labyrinth.

**Fig. 2:**
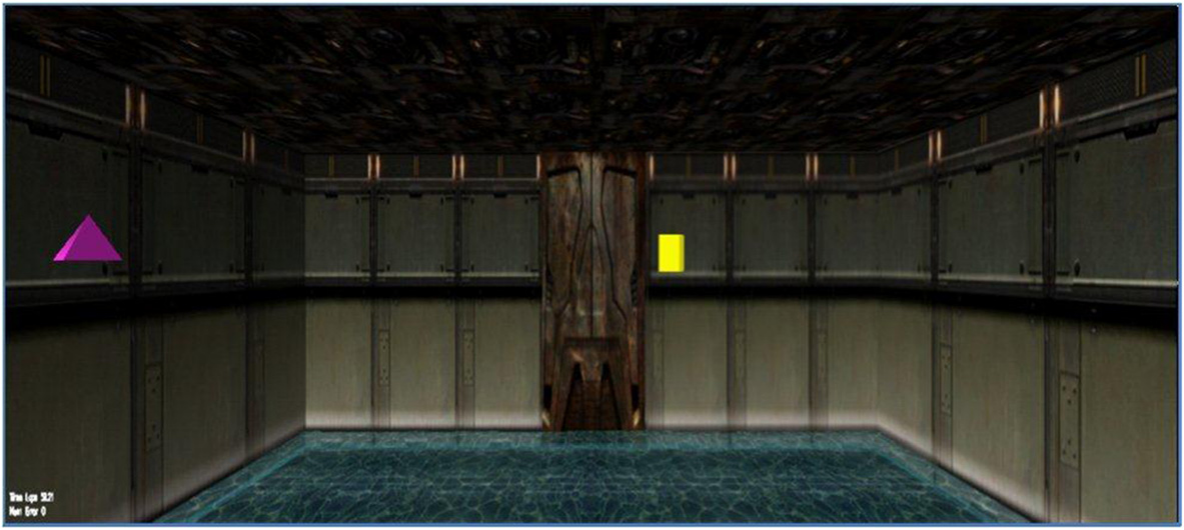
Screenshot of the Spatial Orientation and Learning Labyrinth

In a pre-test, candidates had to navigate a labyrinth and to find their way out by learning the alleys, crossings and dead ends. As this was found to be too difficult and frustrating, geometric icons were established as information signs at the doors (see picture 2). Aside from having to learn the alleys, dead ends, etc., participants have to identify which symbol or information sign indicates the correct way out. The task was to identify three of nine correct symbols. Both spatial orientation and learning aptitude were measured in this labyrinth. In order to enhance the stress level and the urgency of fast task completion, virtual water was flooding into in the labyrinth continuously with a loud sound. The water level stopped at 1.40 m, so that no one had the impression that he or she might drown. Before starting, the participants got the following information: Fig. 2

*“Find your way out of the labyrinth. 9 different symbols will be presented to you: three different colors and three different shapes, three symbols are correct, six symbols are wrong. The task is to memorize the symbols and find the right way out. Remember: six symbols lead into a blind alley and three show the right way!”*

There are different error indices. First, it was automatically counted as an error when a subject opened a door with an already tested and incorrect symbol for a second time. In addition, it was counted as an error when a previously entered, correct door was passed again in the opposite direction. Walking back without any negative feedback is considered as an indicator of losing orientation in the labyrinth. This error forms the ‘loss of orientation’ index. In total there were 24 rooms. Every symbol was evenly distributed throughout the test.

#### **•**Cognitive flexibility test in Virtual Reality (VR)

**Fig. 3:**
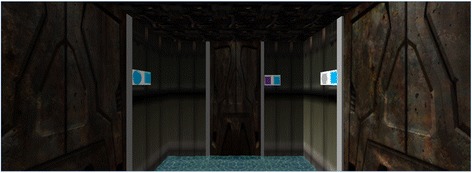
Screenshot of the Cognitive Flexibility Labyrinth

The second, newly developed cognitive test measures cognitive flexibility. Based on the Wisconsin Card Sorting Test (Berg 1948) a labyrinth was constructed to measure cognitive flexibility. Again subjects had to find their way out of a labyrinth by choosing one of three doors to enter the next room. Each door is marked by a different symbol (see Fig. 3).

The symbols consist of two shapes representing one of four possible, yet equal combinations of color and form: (1) equal color and different form, (2) equal form and different color, (3) equal color and equal form, (4) unequal color and unequal form (in total exist three different shapes and three different colors). The current correct principle was either equal color or equal form and has to be figured out by the participants in order to avoid aversive, negative feedback while moving through the labyrinth. The solution was the following: when the participants have identified the right criteria (equal color of the symbols - see left door), the ‘correct answer’ switched after 5 correct trials. So the participants had to decipher that now, the correct answer was ‘same form’ (see door in the middle). Again, after five correct trials the ‘correct answer’ switched back to ‘same color’ followed by another switch after 5 correct choices. The task can be summarized as that after five correct choices in a row, the current principle which is invisible for the subject, changed. Based on the negative feedback, the participant had to deduce the change and determine the new principle. Impaired cognitive flexibility was tested in this paradigm. The respective error is called: ‘perseveration error’, which means that a subject is sticking to a previously correct response and is unable to learn and adapt to a new concept. Beside this, another error measured was ‘arbitrary changes’, which means that an individual switches category without negative feedback which implies that they have done so for no reason. Another error index is ‘number of rooms’ a participant crosses before figuring out the underlying concept for the first time. The error index ‘number of concept changes’ indicated how often a subject identifies the ‘change after 5 correct trials’ during the whole test.

In total there were 40 rooms. There was no fixed ground layout like in the ‘Memory Labyrinth’. No matter which door the participants choose, the programmed algorithm generated the next room. Again, the basic idea was to test how long people stick to their ‘wrong’ criteria even if it was previously shown to be correct, and determine when they start to look for a new solution. Participants received feedback after every door in the form of a red or green flashlight plus positive or negative sounds. The instruction was:

*“Please find your way out of the labyrinth! You will receive feedback if you made the right decision after each room.”*

#### **•**Navigation through the VR labyrinths

**Fig. 4:**
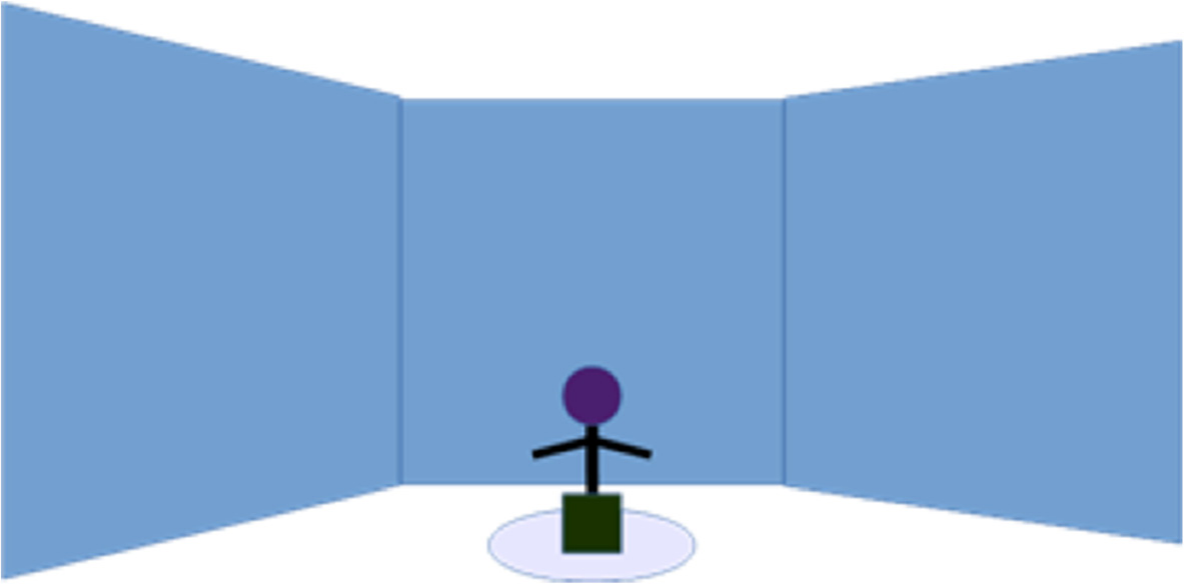
Set up for the participants

The TSST as well as the labyrinths took place in a 3-walled Cave-like system also known as a 3D Powerwall. This is approximately a 3 m cubed area, with projection screens on the three walls providing a stereo view. Participants were standing approximately 2 m away from the three screens (see Fig. 4).

The study was carried out with one participant at a time, using a Sony PlayStation^©^ for navigation. After 1–2 min of training in a ‘neutral labyrinth’ with the game pad, none of the participants showed problems with navigation.

#### **•**Subjective Stress level

Stress was measured on a subjective level via paper and pencil questionnaire. Six questions have been developed and given shortly after the presentation of the individual speech in front of the VR auditory (TSST). The first three questions focused on the subjective stress level and the last three on the evaluation of the VR Task. The authors are convinced that rating an event as “unexpected” leads to similar, but weaker, emotional response than rating an event as “stressful” or “exhausting”. The meaning behind the question “unexpected” can be interpreted as a mild form of stress. The authors also assume that an “unexpected” event could create awareness whereas “stress” might lead towards a stronger reaction. These different aspects of stress (“unexpected”, “stressful” and “exhausting”) are deduced from the model by Lazaruas. These three adjectives reflect the chronological order of stress development. The first step in the stress process is that an expectation of an upcoming event is not fulfilled. In this example, it would be that the participants are surprised that they have to give a free speech. If the outcome of this first evaluation process is “unexpected”, a positive or negative surprise effect is triggered which can be regarded as the beginning of stress. The authors as well as the founders of TSST (Kirschbaum et al. 1993) worked with the assumption that giving a speech is not a stress-free, positive activity for most people. So, rating the event as “unexpected” implies mild stress. Following Lazarus and Folkman 1984, “stress” is the final result of a more elaborated evaluation process. After detecting that an event was unexpected, it has to be clarified if the event is harmful or detrimental to the individual and if enough resources are available to the individual to alleviate the stress. The outcome of this combined evaluation leads to the actual stress reaction. When a situation has been mastered, the individual might feel exhausted if the requirements to alleviate the stressor almost exceeded the capacity of the individual to solve the problem. To sum it up, all three items measure different chronological stages and different levels of intensity of the stress process.

The questions asked are as follows:

- Was the task unexpected?

- Were you stressed?

- Was the task exhausting?

- Did you have a similar feeling as in a lecture auditorium?

- Was the task real?

- Were you content with your public speech?

#### **•**Physiological stress level

A variety of authors have shown that an increasing heart rate in conjunction with other physiological parameters such as perspiration, respiration and blood pressure fluctuations are closely correlated with stress (Healey and Picard 2005; Bassett et al. 1987) and can be evoked with the TSST procedure Oldehinkel et al. 2011.

Mean heart rate responses were measured for a pre-stress period of ten minutes duration while the participant began reading a neutral text. They were given a two minute preparation period, the speech task for five minutes, and a post-stress period of ten minutes duration where they returned to reading a neutral text. Heart rate values within each period were averaged to generate the mean.

##### Consent

Before starting, informed consent was obtained from each participant following the “Ethical Principles of Psychologists and Code of Conduct” according to the American Psychological 2010. Each participant was provided written information that described the purpose of the research, expected duration, and procedures, possible risks, discomfort, adverse effects, and side effects including motion sickness. Also, a description of any benefits to the participant or to others which may reasonably be expected from the research was included. Explanations on confidentiality and limits of the data, as well as the participant’s right to decline to participate and to withdraw from the research once participation has begun were given.

### Design

After receiving the permission of the Ethics Committee of the University of Basel (no. 108/10), a first contact with the subjects and exclusion of anyone with severe/acute psychiatric illness occurred. No subject was excluded from the study due to a psychiatric illness. Then the subjects received a date for testing in the VR laboratory in the Psychiatric University Clinic in Basel. After arriving in the clinic, the ECG (Electrocardiogram) recording system was applied to the participants and they began to read a neutral text in order to calm down and get accustomed to the VR environment. Later they received an introduction to TSST (two minutes preparation time). After their performance of a 5 min public speech, they had to answer the subjective stress questionnaire. Then they had to perform the two different labyrinths starting with the ‘Memory Labyrinth’ followed by the ‘Cognitive Flexibility Labyrinth’ (Wisconsin Labyrinth). Two participants left the study due to simulator sickness.

#### Study procedure for ECG recording

To estimate autonomic nervous system reactivity during the study protocol, a standard three-lead ECG signal was digitally recorded with a sample rate of 1000 Hz employing a BrainAmp ExG bio signal amplifier system (Brain Products, Munich, Germany). Inter-beat-interval times (RR) were derived from the raw digital signal by means of a robust peak detection algorithm, implemented in MATLAB (MathWorks, Inc., Natick MA, USA). Ectopic heart beats and passages of disturbed ECG signal due to movement artifacts were deleted upon visual inspection. The missing RR intervals in the resulting gaps were estimated using a cubic Hermite spline interpolation technique.

### Hypothesis

1. The virtual reality environment (public speech) is effective enough to induce stress. The effect shall be documented via subjective evaluation using a questionnaire and via physiological response. During preparation of the TSST phase, the speech task and the two cognitive labyrinth tasks, heart rates should be higher than during post-stress periods.
2. Spatial orientation and learning aptitude will deteriorate under stress. Performance is measured by number of errors in the ‘Memory Labyrinth’.
3. Stress impairs cognitive flexibility. Performance is measured by number of errors in the ‘Cognitive Flexibility Labyrinth’ (Wisconsin Labyrinth).

### Statistical analysis

SPSS 19 has been used to conduct all statistical analyses. Correlations were analyzed using Kendall Tau Correlation. One-tailed tests were performed with p set to 0.05 for all analyses.

Heart rate (HR) was calculated from the RR time values and averaged across the TSST- speech and the post-stress period. Autonomic nervous system reactivity was defined as the deviation of HR during TSST- speech condition from post-stress baseline measurement. A repeated-measures *t*-test was used to analyze differences. As it was an explorative study, so no a priori power analysis has been performed to set sample size.

## Results

Part one of hypothesis 1 was confirmed. The virtual stress task, TSST - Trier Social Stress Test, was found to be effective in inducing stress on a subjective level. The average score (on a 1–8 Likert scale) for the question “Were you stressed?” was 5.7 and even 7.1 for the question “Was the task unexpected?”. Another finding was that most people considered the task as real and authentic. The question “Did you have a similar feeling as in a lecture auditorium?” was answered with an average score of 5.3, and the question “Was the task real?” was answered with an average score of 5.8. Interestingly, most people were not satisfied with their speech performance (average 3.6). Table 1

**Table 1:** Mean scores for subjective measurement of stress and evaluation of the VR Task (1–8 Likert scale)

Questions	Average score	Standard Deviation	Lowest score	Highest score
Was the task exhausting?	5.2	1.57	2	7
Was the task unexpected?	7.1	1.60	4	8
Were you stressed?	5.7	1.36	3	7
Did you have a similar feeling as in a lecture auditorium?	5.3	2.10	1	8
Was the task real?	5.8	2.13	1	8
Were you content with your speech?	3.6	1.87	3	8

The second portion of hypothesis 1 was also confirmed. Results showed that the heart rate differed significantly between the post-stress period and the instruction, the preparation and the free speech portions of the experiment. Besides that, participants’ heartbeats differed also significantly between the post-stress period and the two labyrinth conditions. This correlates with the participants’ objective perception of being stressed during all relevant phases of the study. Table 2

**Table 2:** Mean heart rate during post-stress period compared with different TSST phases (T-tests for dependent samples)

	Mean	Std.	T	df	R	Cohen’s d
Post-stress period vs:	70.05	9.07				
Instructions for TSST	82.53	14.89	−7.09***	24	0.84	0.81
Preparation time TSST	85.25	16.70	−6.69***	24	0.77	0.91
Free speech TSST	89.17	18.18	−7.17***	24	0.71	1.09
Memory Labyrinth	76.68	12.45	−4.49***	24	0.81	0.56
Wisconsin Labyrinth	73.80	12.00	−3.404***	24	0.90	0.30

(***p < 0.001, **p < 0.01,'*p < 0.05)

Hypothesis 2 was confirmed. There was a significant positive correlation (r = 0.35) between question number 3 “Were you stressed?” and the error index ‘loss of orientation’. This error indicated that a participant kept ignoring positive and negative feedback in the ‘Memory Labyrinth’. So, if participants perceived stress during the free speech, they presented significantly more errors in the ‘Memory Labyrinth’ *(p* < 0.03) afterwards.

The average error was 23 and it took the participants on average 900 s, nearly 15 min, to complete the Memory Labyrinth. Table 3

**Table 3:** Correlation between subjective measurement of stress and Error index ‘Loss of orientation’

Correlation	Exhausted	Unexpected	Stressed	Feeling	Real	Content
Errors in ‘Memory Labyrinth’	.17	.03	.35*	.12	.14	-.03

(***p < 0.001, **p < 0.01,'*p < 0.05)

Hypothesis 3 was not confirmed. There was a significant negative correlation (*r* = −0.31) between question number 2 “Was the task unexpected?” and the number of errors in the Cognitive Flexibility Labyrinth. If a participant stated that the TSST free speech was unexpected, which indicated mild stress, he presented significantly fewer arbitrary category changes in the Cognitive Flexibility Labyrinth (*p* < 0.05.) afterwards. The average error was 12 and it took the participants on average almost 800 s or approximately 13 min to complete the labyrinth. Table 4

**Table 4:** Correlation between subjective measurement of stress and Error index ‘Arbitrary changes’

Correlation	Exhausted	Unexpected	Stressed	Feeling	Real	Content
Total number of Arbitrary changes in ‘Cognitive Flexibility Labyrinth’	-.07	−.31*	−.02	−.13	.14	.17

(***p < 0.001, **p < 0.01,'*p < 0.05)

## Discussion

The aim of the study was to test cognitive function and decision making in a stressful situation. Learning aptitude, spatial orientation as well as cognitive flexibility are considered to be basic cognitive functions. These basic components were operationalized by constructing and programming two virtual labyrinths. In the first labyrinth or Memory Labyrinth, the ground or spatial layout of a labyrinth had to be figured out and geometric icons had to be identified. This reflected learning aptitude in that to be successful, the participant had to correctly identify exit signs. In a second virtual labyrinth, the Cognitive Flexibility Labyrinth, cognitive flexibility was tested. Participants had to demonstrate their mental flexibility by identifying the changing concept of the correct exit door. In order to induce stress, the Trier Social Stress Test (TSST) was given as a virtual reality scenario, the free ‘job interview’ speech in front of 80 avatars. After the speaking portion of the experiment was completed, the participants were asked if they would describe the experience thus far as “stressful”, “unexpected” or “exhausting”.

The findings suggest that the virtual Trier Social Stress Test was tested successfully to induce stress (Hyp.1). On a 1–8 Likert scale, participants reported they were stressed (average score: 5.7). The highest score was 7.1 for the question: “Was the task unexpected?”. The authors are convinced that rating an event as “unexpected” leads to similar, but weaker, emotional response than rating an event as “stressful”.

It was also found that participants regarded the virtual TSST as real and authentic experience, and expressed that they felt present in the VR scenario. The question “Was the task real?” achieved an average score of 5.8 (on a 1–8 Likert scale). The subjects had somehow similar feelings in the virtual lecture hall as being in a real lecture hall. The authors draw the conclusion that for future research, the virtual TSST can be used as a standardized, elegant and cost-effective way to induce psychosocial stress. Subjects had a sufficient feeling of immersion due to the perception of being physically, emotionally and cognitively present in the situation in the virtual lecture hall.

Results also showed that the heart rate differed significantly between the post-stress, instruction, preparation and the free speech periods. This supports that the TSST is not only rated as stressful on a subjective level but also on an objective-physiological level which supports the argument of good external validity.

The second finding was that subjects who experience stress, show a negative cognitive performance for spatial orientation/learning aptitude (hypothesis 2). A positive correlation was found between question number 2: “Were you stressed?” and the error index ‘loss of orientation’, which indicates that the participant did not follow a strategy or learned concept. He walked forwards and backwards (using wrong doors for a second time and ignoring right doors). The more often someone rated the TSST as stressful, the worse their orientation in the labyrinth and the higher their number of mistakes. So, stressed participants had worse spatial orientation and learning aptitude than participants who perceive the TSST as less stressful. Using VR Technology, the results suggest that psychosocial stress lowers the capacity for spatial orientation and learning aptitude.

Hypothesis 3 was not confirmed. When participants stated that the TSST was “unexpected”, they made fewer ‘arbitrary category changes’ in cognitive flexibility. This error index reflects the subject’s ability to build, test and follow a cognitive concept. As stated earlier, the meaning behind the question “unexpected” can be interpreted as a mild form of stress which could create awareness and enable the individual to react reasonably and in a focused manner. The results suggest that subjects who reported that the TSST was “unexpected” were more activated and more focused on the cognitive flexibility task. This is a clear argument against the finding by Alexander et al. 2007 that stress negatively affects cognitive flexibility.

How can these two results be interpreted? On one hand, in an acutely stress situation where the subject has poor coping skills, the performance for spatial orientation and learning aptitude diminishes and people become demotivated, irrational or even afraid. On the other hand, moderate stress, measured with the question 2 “the task was unexpected”, leads to a better performance in cognitive flexibility. The results suggest that different levels of acute psychosocial stressors have individual effects on selected cognitive variables in a normal population. The findings indicate also that both, the VR stress induction scenario using TSST, as well as the VR cognitive tests, are a first successful step towards a better ecological validity in neuropsychological testing.

## Conclusions

The study was set out to explore cognitive functioning under stressful conditions and to enhance the ecological validity of neuropsychological tests. Regarding cognitive functions under stress our results indicate that the ability to learn and apply new information is limited in a stressful situation such as an emergency. For example, this has to be taken into consideration for evacuation planning. Regarding ecological validity, Kang et al. 2008 stated that in order to get a valid impression about the participant’s abilities, it is not sufficient to determine the patient’s best performance on paper and pencil tests given under ideal conditions in a calm and supportive testing environment. Tests that actually evaluate behaviors and cognitive function in simulated daily activities are needed. There is already existing evidence, aside from our study, that VR can be a solution to improve ecological validity (Chaytor et al. 2006, Renaud et al. 2014). Although our results are very promising it remains necessary to carry out future studies where the findings from conventional neuropsychological tests as well as accredited stress questionnaires (for example SAM from Peacock and Wong 1990) are validated with the results of the TSST and the VR labyrinth tests. Further work is also needed to determine if, for example, pre-existing anxiety-related personality traits might interact with induced mental stress to cause changes in learning aptitude, spatial orientation or cognitive flexibility.

## Abbreviations

CPT: Cold Pressor Test
ECG: Electrocardiogram
HPA: Hypothalamic-Pituitary-Adrenal
HR: Heart Rate
RR: RR is a technical/graphical term reflecting the time between two peak points in an electrocardiogram
SAM: Stress Appraisal Measure
SECPT: Socially Evaluated Cold-Pressor Test
TSST: Trier Social Stress Test
VR: Virtual Reality
